# Moxifloxacin-Induced Seizures: A Case Report

**DOI:** 10.7759/cureus.87981

**Published:** 2025-07-15

**Authors:** Joana Cartucho, Bruno Bonito, Bruna Rodrigues Barbosa, Carla Fernandes, Martinho Fernandes

**Affiliations:** 1 Internal Medicine, Unidade Local de Saúde do Arco Ribeirinho, Barreiro, PRT

**Keywords:** adverse drug reaction, drug-induced seizures, fluoroquinolones neurotoxicity, gaba-a antagonism, moxifloxacin, nmda stimulation

## Abstract

Fluoroquinolones are widely used antibiotics due to their broad-spectrum activity and favorable pharmacokinetic properties. However, increasing evidence has raised concerns about their neurotoxic potential, particularly in vulnerable populations.

We present a case of a 63-year-old woman with a history of pulmonary fibrosis and depression, treated chronically with aminophylline and fluvoxamine, who presented with a generalized tonic-clonic seizure after repeated courses of moxifloxacin. No other metabolic, structural, or toxicological causes were identified. Neurological evaluation and imaging were unremarkable. Following discontinuation of moxifloxacin, there was no recurrence of seizure activity.

Although considered safer among the newer fluoroquinolones, moxifloxacin may induce seizures through gamma-aminobutyric acid type A (GABA-A) antagonism and N-methyl-D-aspartate (NMDA) stimulation, particularly in patients with risk factors such as polypharmacy and prolonged exposure. This case highlights the need for increased clinical vigilance even in younger patients without renal or hepatic impairment.

Moxifloxacin-associated seizures, though rare, must be considered in the differential diagnosis of new-onset seizures, especially in patients exposed to prolonged or repeated fluoroquinolone therapy.

## Introduction

Fluoroquinolones are widely used antimicrobials due to their broad-spectrum activity and favorable pharmacokinetics. However, their central nervous system (CNS) toxicity, though uncommon, has emerged as a critical safety concern. While seizures are a known but rare adverse effect, moxifloxacin has been considered to have low neurotoxicity, particularly among the newer agents [1-3]. Recent case reports challenge this assumption, warranting a closer examination of risk factors and clinical outcomes associated with its use [4-7]. In this context, the authors report a clinical case of seizure likely associated with moxifloxacin use, to raise awareness of this potential adverse effect even in patients without typical risk factors. Although seizures associated with moxifloxacin are rare, their potentially life-threatening nature justifies increased clinical awareness and reporting.

## Case presentation

A 63-year-old woman with a history of pulmonary fibrosis and depression, chronically treated with nintedanib (100 mg once daily), aminophylline (100 mg twice daily), and fluvoxamine (100 mg once daily), presented to the emergency department following a generalized tonic-clonic seizure. Family members reported that the patient had been in her usual state of health until approximately three weeks prior, when she began experiencing progressive anxiety and terminal insomnia. These symptoms coincided with repeated courses of moxifloxacin prescribed for respiratory tract infections. In the 48 hours before admission, she developed intermittent apathy.

On arrival, her Glasgow Coma Scale score was 8. She exhibited lateral tongue biting, roving eye movements, and tonic activity in the left upper limb. Vital signs included blood pressure of 99/61 mmHg, heart rate of 89 bpm, peripheral oxygen saturation of 87% on high-flow oxygen via non-rebreather mask (fraction of inspired oxygen [FiO₂] 100%), temperature of 36.9 °C, and a body mass index (BMI) of 17.7 kg/m².

An arterial blood gas collected before intubation (FiO₂ 100%) revealed significant respiratory acidosis and hyperlactatemia (Table 1). 

**Table 1:** Arterial blood gas values performed before intubation while the patient was receiving 100% FiO₂.

Parameter	Patient value	Reference range
pH	7.15	7.35-7.45
PaCO₂ (mmHg)	70	35-45
PaO₂ (mmHg)	62	80-100
HCO₃⁻ (mmol/L)	23	22-26
Lactate (mmol/L)	6.3	<2.2

Laboratory findings were notable for leukocytosis, hyperglycemia, and slightly elevated lactate dehydrogenase, while renal, hepatic, and coagulation parameters were within normal limits (Table 2).

**Table 2:** Summary of the patient’s blood test results on admission.

Parameter	Patient value	Reference range
Hemoglobin (g/dL)	13.7	12.0-15.6
White blood cell count (×10⁹/L)	15.8	3.9-10.2
Neutrophils (×10⁹/L)	12.19	1.50-7.70
Platelets (×10⁹/L)	233	150-400
International normalized ratio	0.87	-
Activated partial thromboplastin time (seconds)	26.9	23.4-35.4
Glucose (mg/dL)	210	60-109
Urea (mg/dL)	18.1	7.9-20.9
Creatinine (mmol/L)	0.65	0.55-1.02
Sodium (mmol/L)	140	136-146
Potassium (mmol/L)	4.0	4.0-5.1
Chloride (mmol/L)	104	101-109
Calcium (mg/dL)	8.1	8.8-10.6
Aspartate aminotransferase (U/L)	30	<31
Alanine aminotransferase (U/L)	27	<34
Alkaline phosphatase (U/L)	60	30-120
Gamma-glutamyltransferase (U/L)	28	<38
Total bilirubin (mg/dL)	0.4	0.2-1.2
Lactate dehydrogenase (U/L)	296	<247
Creatine kinase (U/L)	76	<145
C-reactive protein (mg/dL)	0.10	<0.50
Serum ethanol	<0.10	-
Thyroid-stimulating hormone (uUI/mL)	4.3	0.4-4.0
Free thyroxine (ng/dL)	0.78	0.7-1.5

A toxicology screen was performed, revealing positive urinary benzodiazepines, likely related to procedural sedation. No other substances were detected (Table 3).

**Table 3:** Drug screening panel results from serum and urine samples. THC, tetrahydrocannabinol

Substance	Sample	Patient value	Reference range
Benzodiazepines	Serum	Negative	Negative
Benzodiazepines	Urine	>1,000 ng/mL	<200 ng/mL
Barbiturates	Urine	Not detected	Negative
Cannabinoids (THC)	Urine	Not detected	Negative
Cocaine	Urine	Not detected	Negative
Opiates	Urine	Not detected	Negative
Amphetamines/Methamphetamines	Urine	Not detected	Negative

The patient was sedated and intubated. After two hours, sedation was stopped, and she was successfully extubated. At this time, she was somnolent but easily arousable, disoriented, and amnestic for the preceding two days. No focal neurologic deficits were identified. Repeat blood gas post-extubation (2 L/minute O₂ via nasal cannula) showed normalization of parameters (Table 4).

**Table 4:** Post-extubation arterial blood gas performed after extubation while on 2 L/minute oxygen via nasal cannula.

Parameter	Result	Reference range
pH	7.39	7.35-7.45
PaCO₂ (mmHg)	32.7	35-45
PaO₂ (mmHg)	66.0	80-100
HCO₃⁻ (mmol/L)	20.0	22-26
Lactate (mmol/L)	1.3	<2.2

Extensive investigations were conducted to exclude infectious, structural, and metabolic causes. Cerebrospinal fluid (CSF) analysis, including cytochemical, microbiologic, and polymerase chain reaction (PCR) testing for common neurotropic pathogens, was unremarkable (Table 5). The patient was also tested for a broad panel of antineuronal antibodies, all of which returned negative, ruling out autoimmune neurological disorders.

**Table 5:** Cerebrospinal fluid analysis, including cytochemical, microbiological, and molecular testing. PCR, polymerase chain reaction

Cytochemical analysis		
Parameter	Patient value	Reference range
Proteins (mg/dL)	28	15-40
Glucose (mg/dL)	76	40-76
Chloride (mmol/L)	126	116-122
Lactate dehydrogenase (U/L)	<18	-
Leukocytes (cells/mm³)	<3.0	<3.0
Erythrocytes (cells/mm³)	<3	<3
Microbiological analysis		
Direct Gram Strain	Rare leukocytes observed, no bacteria or yeast-like fungi detected	
Culture	Negative for aerobic bacteria and yeast-like fungi	
Meningitis/encephalitis PCR panel		
Herpes Simplex Virus 1	Not detected	
Herpes Simplex Virus 2	Not detected	
Enterovirus	Not detected	
Varicella Zoster	Not detected	
Human Herpesvirus 6	Not detected	
Neisseria meningitidis	Not detected	
Streptococcus pneumoniae	Not detected	
Haemophilus influenzae	Not detected	
Escherichia coli K1	Not detected	
Streptococcus agalactiae	Not detected	
Listeria monocytogenes	Not detected	
*Cryptococcus neoformans/Cryptococcus gattii*	Not detected	

**Figure 1:**
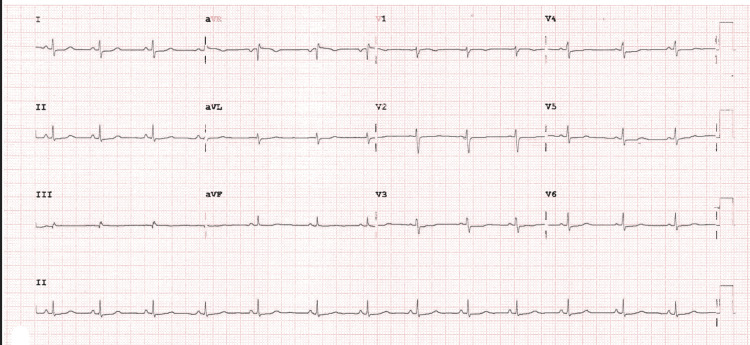
Twelve-lead electrocardiogram. Sinus rhythm with a heart rate of 79 bpm and no significant abnormalities.

**Figure 2:**
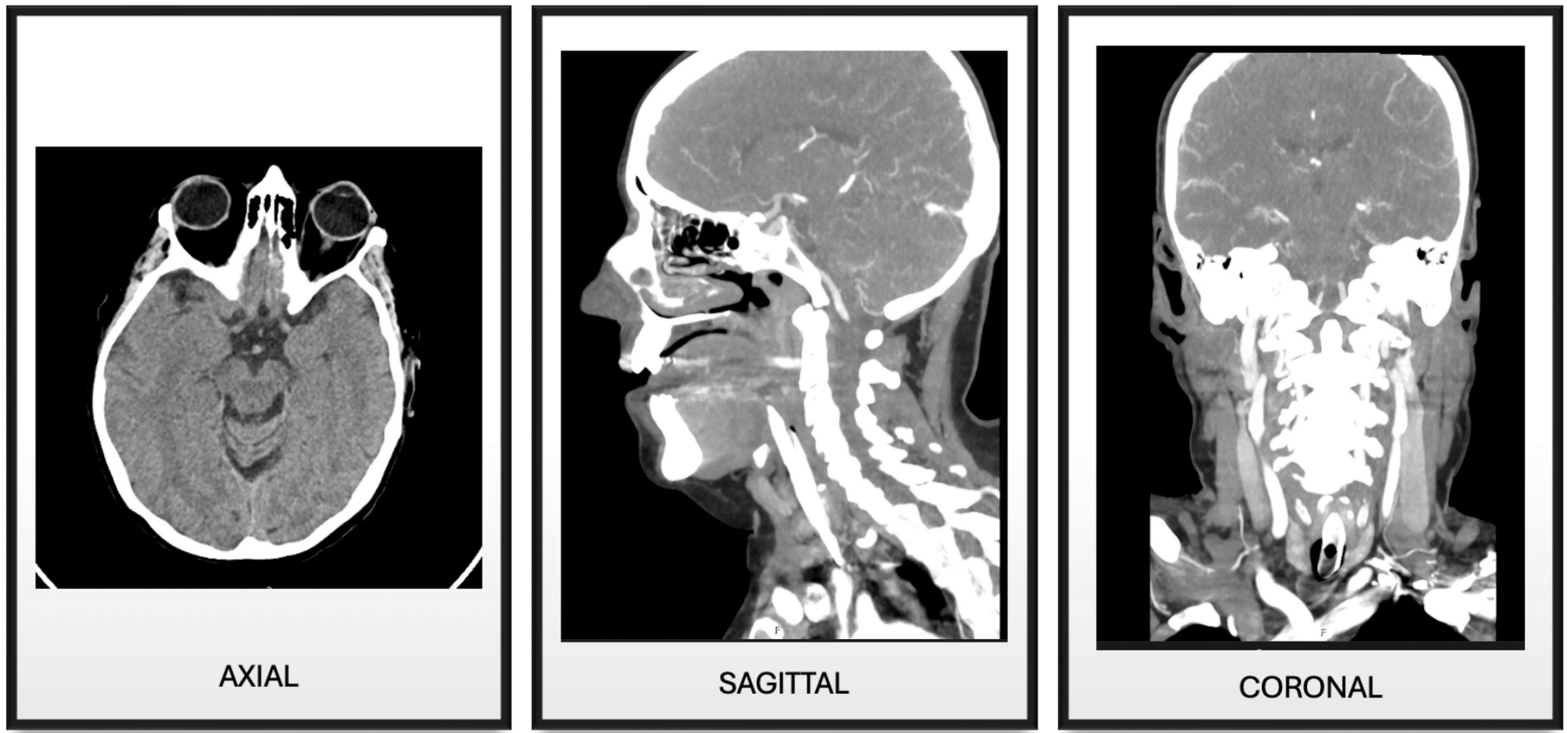
Cranial and cervical computed tomography angiography. No intracranial abnormalities were observed on density-based imaging. No evidence of foramen magnum crowding. The vascular assessment revealed normal patency of the major cervical vessels and skull base arteries.

**Figure 3:**
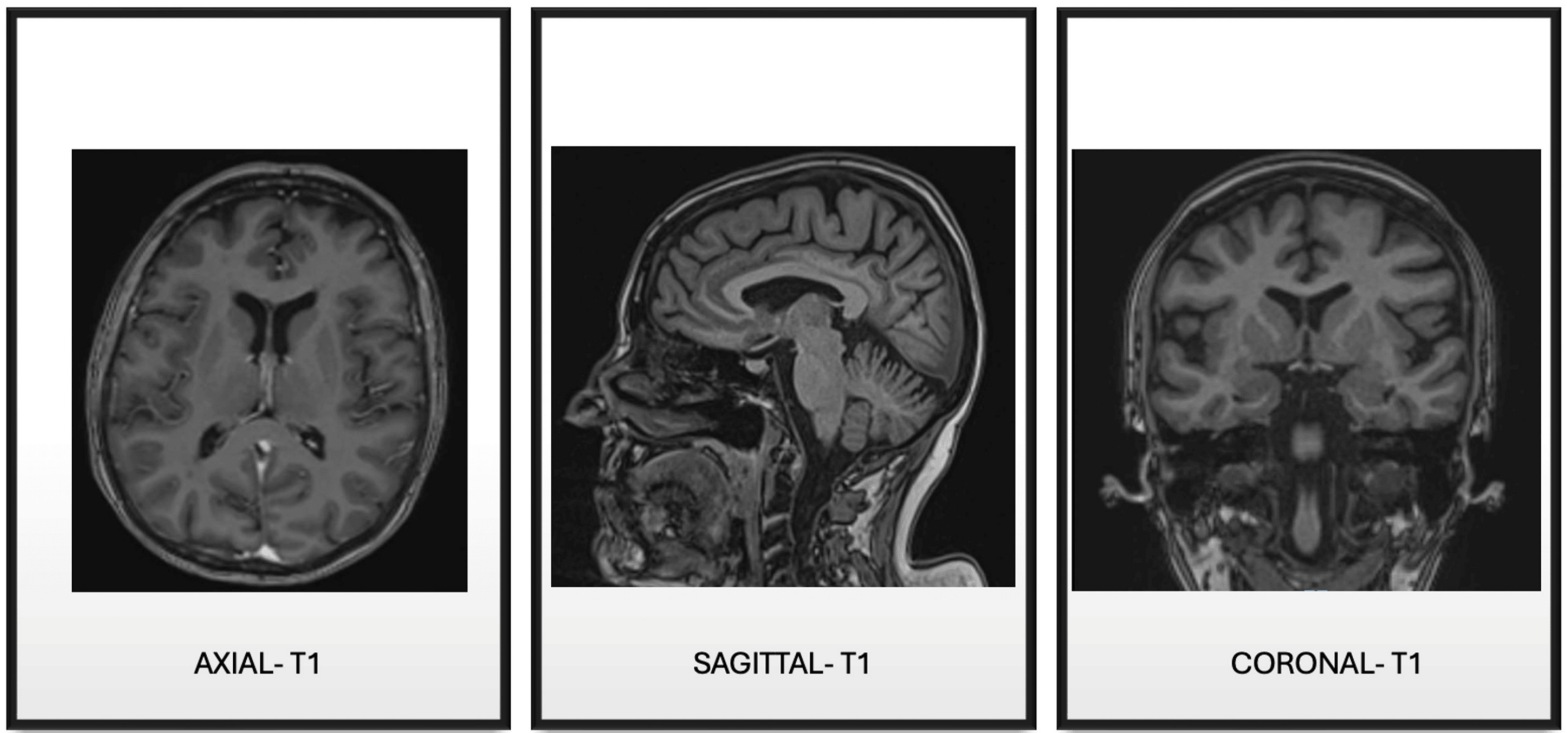
Brain magnetic resonance imaging. No significant age-related abnormalities were identified. No evidence of expansive, vascular, acute, or chronic lesions.

**Figure 4:**
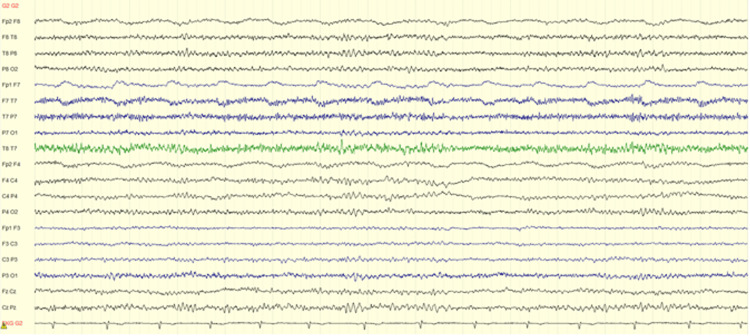
Electroencephalogram. Signs of mild encephalopathy with normal background activity but superimposed intermittent slowing, without epileptiform potentials.

Electrocardiogram (Figure 1), cranial and cervical CT angiography (Figure 2), and brain MRI (Figure 3) revealed no acute findings. Electroencephalogram (Figure 4), however, showed signs of mild encephalopathy, with normal background activity and superimposed intermittent slowing, without epileptiform potentials.

The temporal relationship between moxifloxacin exposure and seizure onset, alongside exclusion of alternative etiologies, supports a probable causal association according to the Naranjo Adverse Drug Reaction Probability Scale. The patient remained stable and seizure-free and was discharged home on day 3 with instructions to avoid future moxifloxacin use. 

## Discussion

CNS adverse effects represent the second most frequent type of fluoroquinolone toxicity, following gastrointestinal complications. Symptoms range from headache and confusion to tremor, psychosis, and seizures. The incidence is estimated at 1%-2% overall, with seizures being rare but potentially fatal events [1,2].

Historically, moxifloxacin was considered to have a lower potential for CNS-related side effects compared to earlier fluoroquinolones [3,4]. However, emerging evidence suggests that moxifloxacin may induce seizures under certain clinical conditions. From the reviewed literature, two clinical case reports stand out for their detailed documentation of seizure onset temporally associated with moxifloxacin administration [5,6].

In the case by Unzurrunzaga et al., a 79-year-old man with a history of seizures developed status epilepticus after four days of oral moxifloxacin for a respiratory infection [5]. The patient had moderate renal impairment and was concurrently taking theophylline, a known proconvulsant. Although moxifloxacin is not traditionally considered to interact with theophylline, the latter may potentiate GABA receptor inhibition, thereby lowering the seizure threshold [5]. The Naranjo probability scale indicated a “probable” relationship between the drug and the seizure event.

Similarly, Shi and Xu reported a 73-year-old woman who experienced a generalized tonic-clonic seizure on the sixth day of intravenous moxifloxacin administration for appendicitis [6]. Despite no history of epilepsy, the patient had a previous cerebrovascular injury, severe renal and hepatic impairment, and electrolyte disturbances (hyponatremia and hypocalcemia), all of which are recognized seizure risk factors. Discontinuation of moxifloxacin led to full recovery without recurrence of seizures.

As with previously reported cases, this case highlights several identifiable risk factors, including chronic use of aminophylline and fluvoxamine, as well as repeated courses of moxifloxacin over three weeks. Notably, the patient differs from most previously described cases by being younger and having no evidence of renal or hepatic impairment. Finally, this case underscores the importance of recognizing early signs of central nervous system involvement, particularly absence seizures, which were overlooked until the classic presentation of a generalized tonic-clonic seizure occurred.

Pharmacologically, fluoroquinolone-induced seizures are thought to result from competitive antagonism at GABA-A receptors, leading to reduced inhibitory neurotransmission, as well as potential stimulation of NMDA receptors - both mechanisms contributing to increased neuronal excitability [7]. The risk of neurotoxicity is influenced by the compound’s lipophilicity and ability to penetrate the CNS, as well as structural modifications at the C-7 position of the quinolone ring [7]. Although moxifloxacin lacks high-risk substitutions, its capacity to cross the blood-brain barrier may still pose a threat in predisposed individuals.

These mechanistic insights help explain why even patients without classical risk factors may still develop CNS toxicity under certain conditions. The updated evidence highlights that moxifloxacin-induced seizures are more likely in the elderly, those with renal impairment, prior CNS disease, or concurrent use of CNS-active drugs. While pharmacokinetics is generally stable across age groups, elderly females may have reduced drug clearance due to lower body weight, potentially increasing systemic exposure [5]. This consideration is particularly relevant to the present case, where, although the patient was younger and had no organ dysfunction, her low body mass index (17.7 kg/m²) may have contributed to increased systemic exposure and neurotoxicity.

## Conclusions

Moxifloxacin, though generally well-tolerated, can induce seizures in vulnerable individuals. The risk appears to be heightened by renal dysfunction, preexisting CNS conditions, electrolyte imbalance, advanced age, and concurrent medications. Clinicians should carefully assess patient history before prescribing fluoroquinolones and maintain vigilance for early signs of neurotoxicity. A personalized risk-benefit analysis is essential, particularly in the elderly or those with comorbidities.
